# An Increased Understanding of Enolate Additions under Mechanochemical Conditions

**DOI:** 10.3390/molecules22050696

**Published:** 2017-04-27

**Authors:** Heather Hopgood, James Mack

**Affiliations:** Department of Chemistry, University of Cincinnati, Cincinnati, OH 45221-0172, USA; hopgoohm@mail.uc.edu

**Keywords:** mechanochemistry, green chemistry, enolate, solvent-free

## Abstract

Very little is known about enolate addition chemistry under solver-free mechanochemical conditions. In this report, we investigated the ability to selectively form products arising from the primary, secondary, and tertiary enolates under solvent-free conditions. Using potassium *tert*-butoxide as the base and primary, secondary, and tertiary electrophiles, we were able to generate various enolate addition products including, 1,3,3,3-tetraphenyl-2,2-dimethyl-1-propanone; a molecule we did not observe under traditional solution-based conditions.

## 1. Introduction

Enolates are a very powerful synthetic tool for the formation of highly electron-rich carbon atoms that can serve as nucleophiles. Unlike a Grignard or other organometallic reagents, however, enolate reactions are not air- or water-sensitive and therefore can be conducted under atmospheric conditions. Surprisingly, given the importance of enolates in organic synthesis, there are very few examples in the literature that are conducted under mechanochemical conditions [1,2,3,4,5,6]. In solution, studies have shown that enolate reactivity is dependent on a few factors. Rappe and Sachs determined that the rate of deprotonation of acetone is faster than that of 3-methyl-2-butanone by a factor of approximately 100; the addition of two methyl groups significantly reduces the availability of the proton for removal [7]. Additionally, enolate reactivity can be affected by both the solvent and the coordinating ion [8]. A study by House et al. indicated that, when using dimethoxyethane, the electron-density on the α-carbon to the carbonyl changes with a different conjugate alkali metal, which affects the amount of C-alkylation observed in the product [9]. Jackman and Lange went on to demonstrate that this is largely due to the formation of ion-pair aggregates. For example, the lithium enolate of isobutyrophenone predominates as a solvated tetramer in dioxolane. These aggregates facilitate C-alkylation, and it was determined that softer leaving groups, such as iodide, are more favorable [10]. This trend has shown to be qualitatively consistent in both the gas phase and in solvent, suggesting that it may not be a solvent-dependent phenomenon [11].

In order to better understand enolate chemistry under solvent-free mechanochemical conditions, we sought to carry out various enolate reactions under these unique conditions. Some enolate reactivity has previously been demonstrated using mechanochemical conditions. Toda et al. reported the mixed aldol condensation of *p*-methylbenzaldehyde with acetophenone in the presence of sodium hydroxide using a mortar and pestle. They found the yield to be higher under solvent-free conditions than when the reaction was conducted in aqueous ethanol [12]. Bolm, Soloshonok, and co-workers developed asymmetric variants of enolate reactions using different catalysts under mechanochemical conditions [6,13]. We previously demonstrated the ability to control kinetic and thermodynamic enolates using mechanochemistry [14].

Although enolate reactions have been shown to be favorable using mechanochemistry, little is known about the addition of electrophiles to enolates under these conditions. In the examples above, additional products were reported leaving the range of enolate reactivity still unclear. In order for a more consistent use of enolate chemistry to be conducted under mechanochemical conditions, these gaps must be addressed.

## 2. Results and Discussion

We started our study by first trying to understand which bases would be best suited for solvent-free enolate addition reactions. Using acetophenone and benzyl bromide, we tested several bases to determine which base gave the highest yield of substitution.

As can be seen from the results in Table 1, typically as base strength increases, so does the overall yield of the reaction. It is interesting to point out that lithium hexamethyldisilazide is a stronger base in solution than potassium *tert*-butoxide, but led to lower enolate addition product under mechanochemical conditions. This is due to the addition of benzyl bromide to the lithium hexamethyldisilazide. Similar to what is observed in solution, the diaddition occurs in higher yield than the mono addition in most cases [15]. From the initial screening of the reaction, potassium *tert*-butoxide gave the highest yield of products, so we chose to use potassium *tert*-butoxide in the subsequent reactions.

**Scheme 1:**
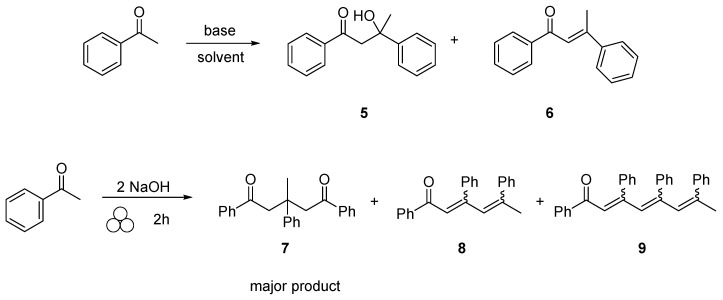
Comparison of acetophenone self-aldol in solution versus mechanochemistry.

Typically in solution, enolate substitution reactions are set up in separate steps to avoid the competing aldol condensation. We wanted to test how prevalent the aldol condensation reaction was in comparison to the substitution reaction under mechanochemical conditions. When we attempted to conduct the enolate reaction using acetophenone stepwise, we observed 3-methyl-1,3,5-triphenyl-1,5-pentadione (**7**). This is believed to come from an initial aldol condensation followed by a Michael addition. In order to determine if we could stop the reaction at the aldol intermediate, we varied the time of the reaction from 2–16 h. At every interval we attempted, we were unable to observe 3-hydroxy-1,3-diphenylbutan-1-one (**5**) or dypnone (**6**) in the reaction mixture. Since the energetics of our mill has been theorized to be similar to 90 °C, it may not be surprising to observe the rapid loss of water under these conditions [16]. However, given the fact that we did not observe the α,β-unsaturated ketone, the 1,4 addition might occur at a much faster rate than the initial aldol condensation. In addition to the 1,5-diketone, we observed other products related to the 1,2 addition of the enolate with initial Michael addition products [17]. The 1,2 addition of the α,β-unsaturated ketone has been observed under solution conditions as well, but only after trapping the α,β-unsaturated ketone with diethylboryl-pivalate [18]. This is in stark contrast to solution, where the aldol condensation of acetophenone gives 3-hydroxy-1,3-diphenylbutan-1-one (**5**) and dypnone (**6**) (Scheme 1) [19].

1,5-Diketones have been used as synthetic intermediates for heterocyclic and polyfunctional compounds which can be useful in coordination chemistry, molecular sensing, catalysis and redox active self-assembly devices [20]. For example, Smith et al. has used 1,5-diketone chemistry for the formation of 2,4,6-triarylpyridines [21]. These precursors are used for supramolecular synthesis for polymers, as well as, in new therapeutic drugs. Some difficulty has been observed with the Michael addition of α,β-unsaturated aryl ketones because of the steric hindrance on both substrates and strong bases are often used with low yield formation. Alternative methods have been explored including metal catalysis [22], enamine catalysis [23], and promotion by barium hydride or barium alkoxides [24]. Therefore, mechanochemistry provides a simple method to gain access to these 1,5-diketones. Given the propensity of enolates to form aldol condensations products very rapidly under milling conditions, we conducted all subsequent enolate addition reactions in one-step instead of the two-step process traditionally executed in solution.

**Figure 1:**
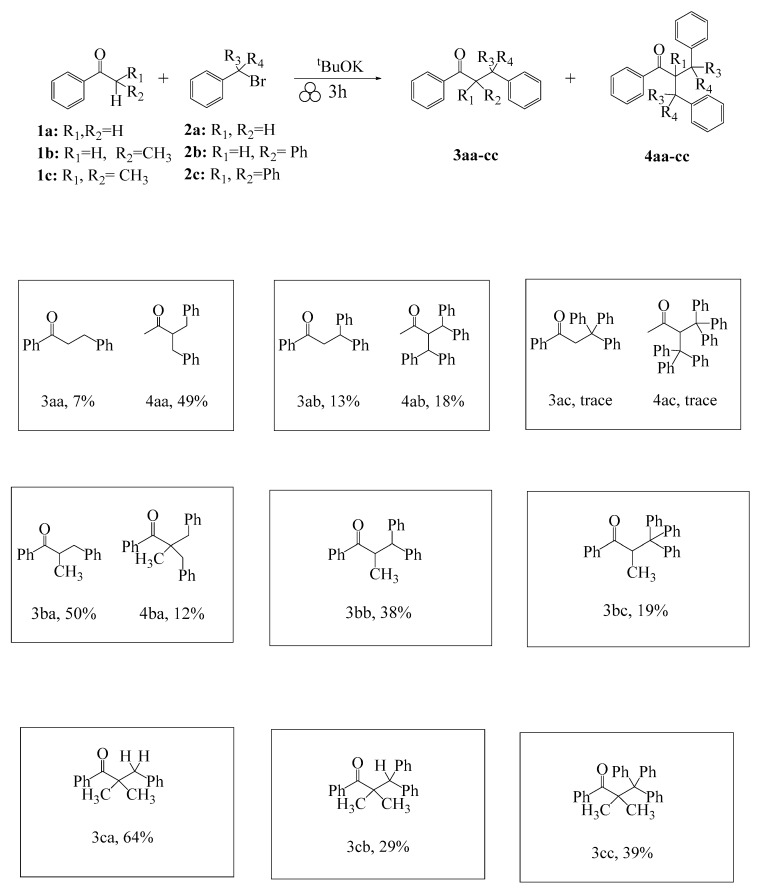
Enolate reactivity results.

With a clearer understanding of the reactivity trends of enolates under mechanochemical conditions, we sought to determine exactly how it affects the productivity of enolate addition reactions. In order to address these questions, a range of enolate reactions was conducted using primary, secondary, and tertiary enolates with primary, secondary, and tertiary alkyl bromides. Potassium *tert*-butoxide was used as the base from the results of the previous study. Furthermore, since these reactions need to be conducted in a one-step fashion, the bulky nature of the *t*-butyl group was expected to decrease the competitive nucleophilic addition reaction by the base. The results of this study are given in Figure 1.

From the given results, it is clear that various competitive reactions are highly influential in the formation of the enolate addition product. When comparing ketone reagents on solid support, Muzart demonstrated the aldol condensation reactivity is highest for acetophenone as compared to more sterically hindered ketones [25]. In addition to the aldol condensation, the base can also act as a nucleophile which leads to additional side products in many of these reactions. We believe strategic control over the steric bulk of the ketone and alkyl halide is key to controlling the yield of mono addition products. In the case of isobutyrophenone (**1c**), which forms a tertiary enolate (entries **3ca**–**3cc**), the presence of the two methyl groups prevents the elimination step needed to form the α,β-unsaturated ketone such that it leads to the highest yield of mono addition product [26].

Additionally, sterically hindered reactions are difficult to conduct under conventional solution-based conditions and often require the use of activated enolates such as enol acetates, enamines, 1,3-dicarbonyls, or silyl enol-ethers. For instance, McGlacken et al. achieved a 15% product yield from the reaction of propiophenone with benzyl bromide through the activation with a chiral imine [27]. Similarly, Maruoka et al. recovered a 73% yield with the activated silyl enol-ether of isobutryophenone and chlorodiphenylmethane through the use of a methylalumoxane catalyst [28]. In this case, both trimethylsilane and the aluminum catalysts were used for the activation of the ketone reagent. To demonstrate the ability to generate sterically crowded molecules via these unique conditions, we were able to synthesize 1,3,3,3-tetraphenyl-2,2-dimethyl-1-propanone (**3cc**) in a 39% yield, a molecule we were unable to observe under solution-based conditions.

**Figure 2:**
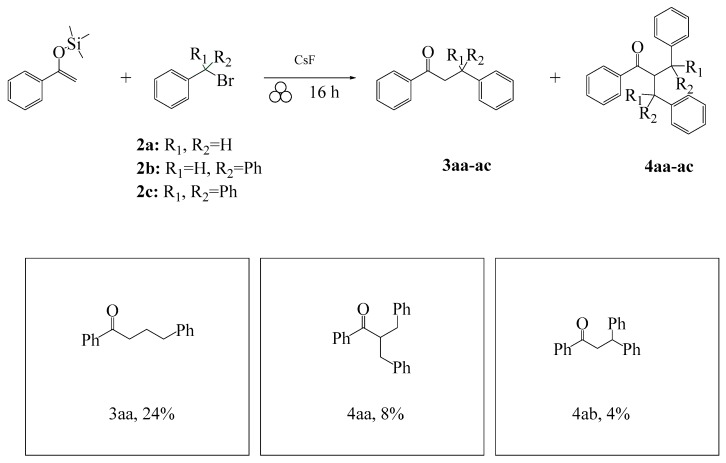
Silyl enol ether reactivity under mechanochemical conditions.

Our previous results suggest competing reactions with primary enolates may ultimately lead to low mono addition product formation. In order to alleviate this problem, the trimethylsilyl-enol ether of acetophenone was generated to determine if these enolate equivalents would perform better under mechanochemical conditions. We milled the silyl enol ether of acetophenone in the presence of cesium fluoride with benzyl bromide, bromodiphenylmethane, and triphenylbromide to compare the reactivity to our earlier experiments. The results of this latter study are given in Figure 2.

As was expected, the trap has enabled a greater selectivity for single-addition products confirming an increase in control of the reactivity. Although no aldol condensation products were observed, the overall yields of the enolate addition products decreased significantly.

## 3. Materials and Methods

General: Mechanochemical milling reactions were carried out in a SPEX 8000D Miller/Mill (Spex Certiprep, Metuchen, NJ, USA) at a frequency of 18 Hz using a custom made stainless steel vial with a single stainless steel ball bearing. ^1^H Nuclear Magnetic Resonance (NMR) spectra were obtained using a Bruker Avance 400 MHz spectrometer (Bruker, Billerica, MA, USA), ^13^C-NMR spectra were recorded at 100 MHz and all chemical shift values are reported in ppm on the δ scale. Gas chromatography–mass spectrometry (GC–MS) data was obtained using a Hewlett-Packard 6890 series GC–MS system (Agilent, Santa Clara, CA, USA) with an HP-5MS, 30 m × 0.25 μm × 0.25 μm column (Agilent). High resolution mass spectral determinations were carried out by using a Micromass Q-TOF-2 Mass Spectrometer (Waters, Milford, MA, USA) in positive mode. Analytical Thin Layer Chromatography (TLC) was performed on silica gel plates using UV light. Flash column chromatography was performed on a CombiFlash Automated Flash Chromatography system (Teledyne Isco, Lincoln, NE, USA) by using RediSep Rf Gold high performance flash columns (fine spherical silica gel 20–40 μm) (Teledyne Isco). Deuterated chloroform was obtained from Cambridge Isotope Laboratories Inc. (Andover, MA, USA) and used without further purification. All reagents were purchased from Acros Organics and used without further purification. Custom-made vials were made by the machine shop at the University of Cincinnati with metal rods purchased from McMaster-Carr (Aurora, OH, USA). Simriz 486 Perfluoroelastomer O-rings (6/16” ID × 7/16” OD × 3/32” width) were purchased from Small Parts Inc. (Logansport, IN, USA). NMR Spectra can be found in Supplementary Materials.

### General Enolate Reaction Procedure

Two millimole ketone, alkyl bromide, and potassium *tert*-butoxide (1:1:1) were added to a custom-made stainless steel vial with a 3/16” stainless steel ball. The stainless steel vial was sealed with the use of a Teflon O-ring and tightened with a vice. Reactions were run for 3 h in a Spex 8000D Mixer/Mill. Upon completion, the reaction mixture was extracted with ethyl acetate and dried onto silica under reduced pressure for flash chromatography separation. Product isolation was achieved with a gradient ramp from 100% heptane to 10% ethyl acetate.

## 4. Conclusions

Enolate reactivity under solvent-free mechanochemistry conditions is quite different from that for solution-based conditions. We have observed that it is best to conduct these reactions in a one-step manner rather than two separate steps as traditionally conducted in solution. This is necessary in order to avoid the competitive aldol condensation reaction. The use of a bulky base limits the competitive reaction of the base reacting with the electrophile. Although there are some limitations with conducting enolate reactions under solvent-free conditions, these conditions are beneficial for tertiary enolates, and the formation of sterically hindered products such as 1,3,3,3-tetraphenyl-2,2-dimethyl-1-propanone (**3cc**). Ball milling can be an excellent asset for the reduction of solvent waste and the generation of more environmentally benign reactions. However, in order to achieve these goals, we must first understand the nuances of this process.

## Figures and Tables

**Table 1:**
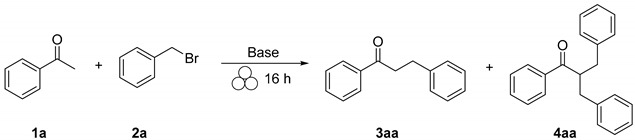
Comparison of bases in enolate reactivity.

Entry	Base	Yield	Product Ratio 3aa/4aa
1	*^t^*BuOK	94%	12/88
2	NaNH_2_	91%	12/88
3	KOH	78%	10/90
4	LiHMDS	52%	42/58
5	NaOH	11%	36/64
6	CS_2_CO_3_	3%	100/0

LiHMDS: lithium bis(trimethylsilyl)amide.

## Supplementary Materials

Supplementary materials are available online.
