# Supplementary material for: An Increased Understanding of Enolate Additions under Mechanochemical Conditions
Source: Molecules. 2017 Apr 27;22(5):696. doi: 10.3390/molecules22050696 (PMC6154340; doi:10.3390/molecules22050696)
Supplement: Supplementary file 1 [file molecules-22-00696-s001.pdf]

Supplementary Materials: Increased understanding of enolate additions under mechanochemical conditions

Heather Hopgood, and James Mack*

1. Spectral Data of selected compounds

^{1}H-NMR (400 MHz, Chloroform-*d*) δ 7.93 (d, *J* = 7.2 Hz, 2H), 7.55 (d, *J* = 7.4 Hz, 1H), 7.43 (t, *J* = 7.6 Hz, 2H), 7.27 (m, 7H), 7.17 (dt, *J* = 8.8, 4.5 Hz, 3H), 4.83 (t, *J* = 7.3 Hz, 1H), 3.74 (d, *J* = 7.3 Hz, 2H). same as reported in Iwai, T.; Tanaka, R.; Sawamura, M., Synthesis, Coordination Properties, and Catalytic Application of Triarylmethane-Monophosphines. *Organometallics* **2016,** *35* (23), 3959-3969.

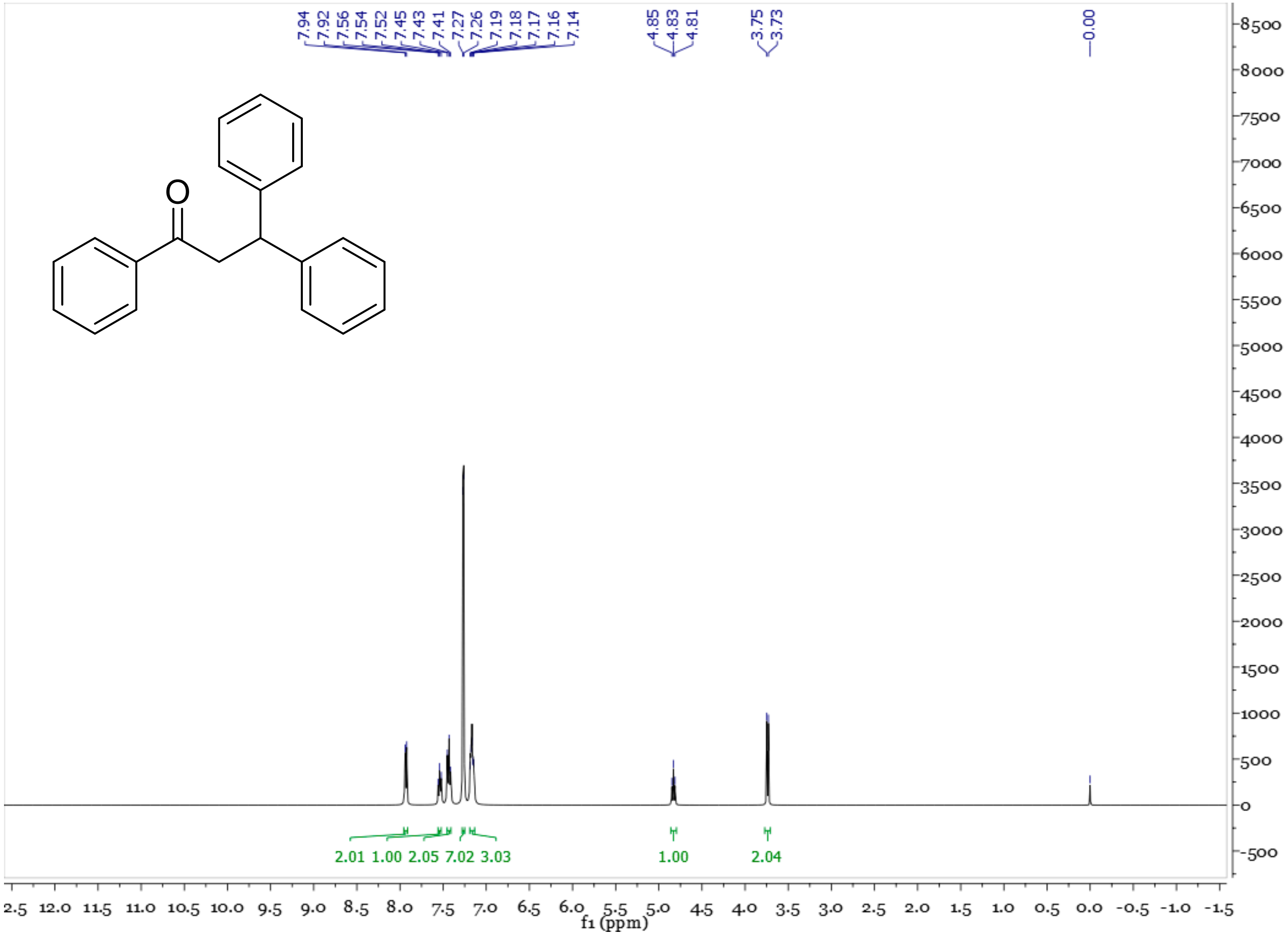

^{13}C NMR ^{13}C NMR (101 MHz, Chloroform-*d*) δ 197.99, 144.15, 137.06, 133.09, 128.61, 128.57, 128.06, 127.85, 126.39, 45.93, 44.74. same as reported in Iwai, T.; Tanaka, R.; Sawamura, M., Synthesis, Coordination Properties, and Catalytic Application of Triarylmethane-Monophosphines. *Organometallics* **2016,** *35* (23), 3959-3969.

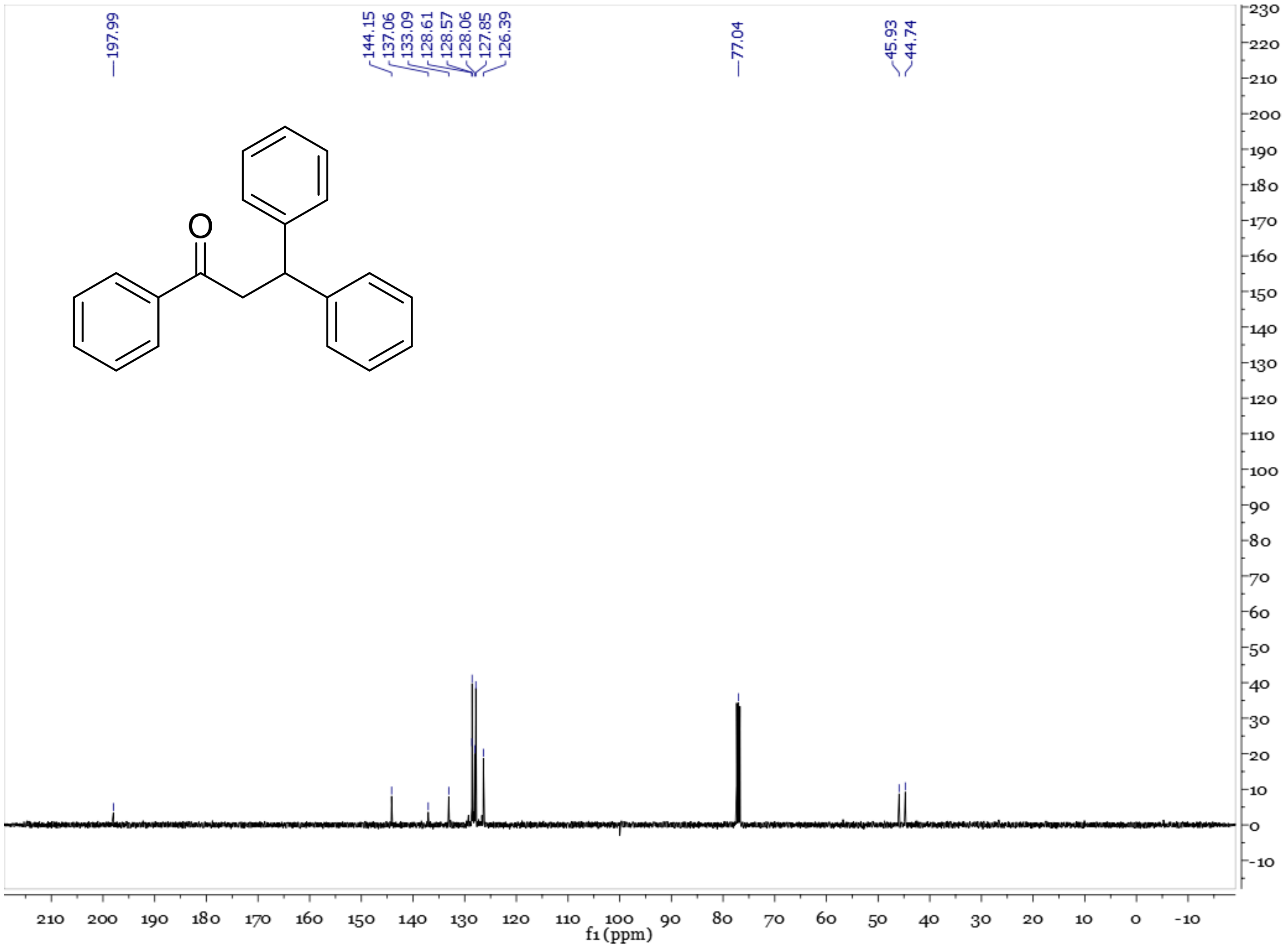

di-addition **^{1}H-NMR** (400 MHz, Chloroform-*d*) δ 7.44 (d, *J* = 7.5 Hz, 2H), 7.33 – 7.27 (m, 2H), 7.22 (d, *J* = 7.5 Hz, 4H), 7.15 (d, *J* = 7.5 Hz, 5H), 7.06-7.12 (m, 8H), 7.05 – 7.00 (m, 4H), 5.26 – 5.20 (m, 1H), 4.45 (d, *J* = 8.9 Hz, 2H). IR (ATR): 3085.8, 3061.1, 3027.4, 2924.3, 2854.9, 1675.4, 1493.8, 728.8, 698.5 cm^{-1} HRMS (ESI) m/z: [M + Na]+ Calcd for $(C_{34}H_{28}O)Na^+$ 475.2032; Found 475.2041. colorless viscous oil

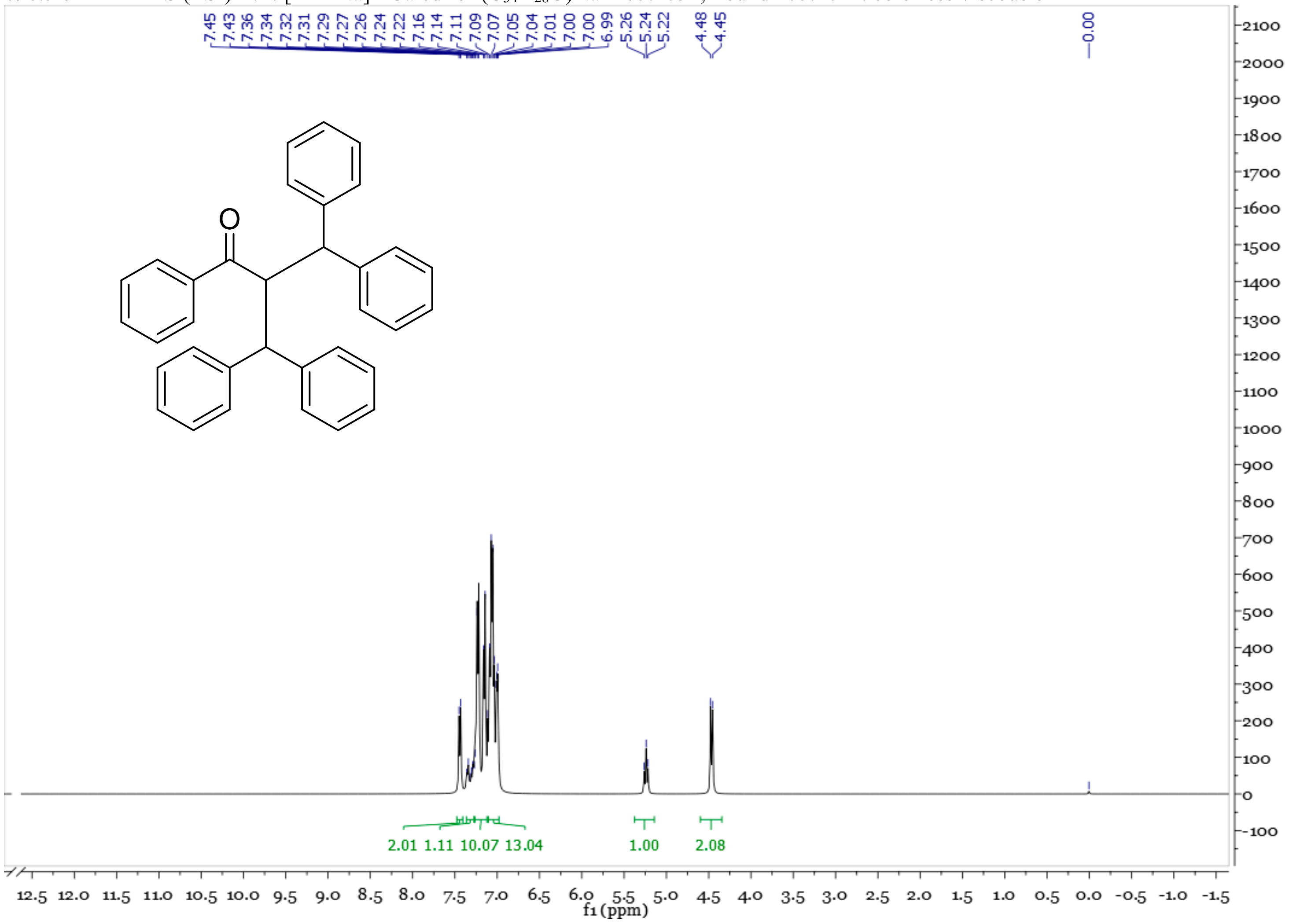

di-addition ^{13}C NMR (101 MHz, $CDCl_3$) δ 203.03 142.43, 141.51, 139.63, 132.03, 129.21, 128.70, 128.15, 128.01, 127.87, 126.43, 126.36, 55.23, 54.53.

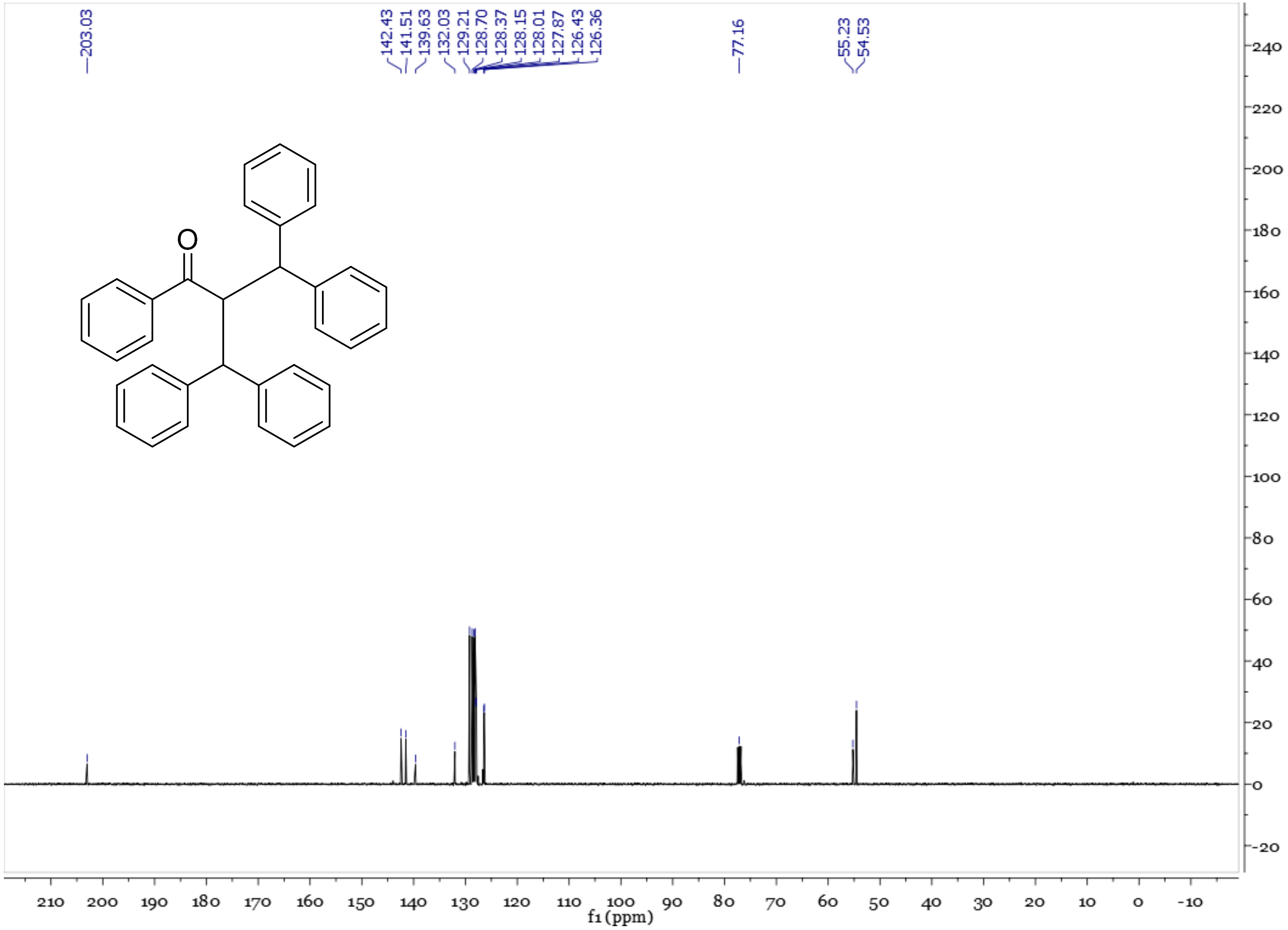

^{1}H-NMR ^{1}H NMR (400 MHz, Chloroform-*d*) δ 7.83 (d, *J* = 7.2 Hz, 2H), 7.50 (t, *J* = 7.4 Hz, 1H), 7.38 (t, *J* = 7.7 Hz, 2H), 7.29 (d, *J* = 5.3 Hz, 3H), 7.26- 7.16 (m, 12H), 4.45 (s, 2H). 1. Same as reported in Koppolu, S. R.; Naveen, N.; Balamurugan, R., Triflic Acid Promoted Direct α-Alkylation of Unactivated Ketones Using Benzylic Alcohols via in Situ Formed Acetals. *J. Org. Chem.* **2014,** *79* (13), 6069-6078.

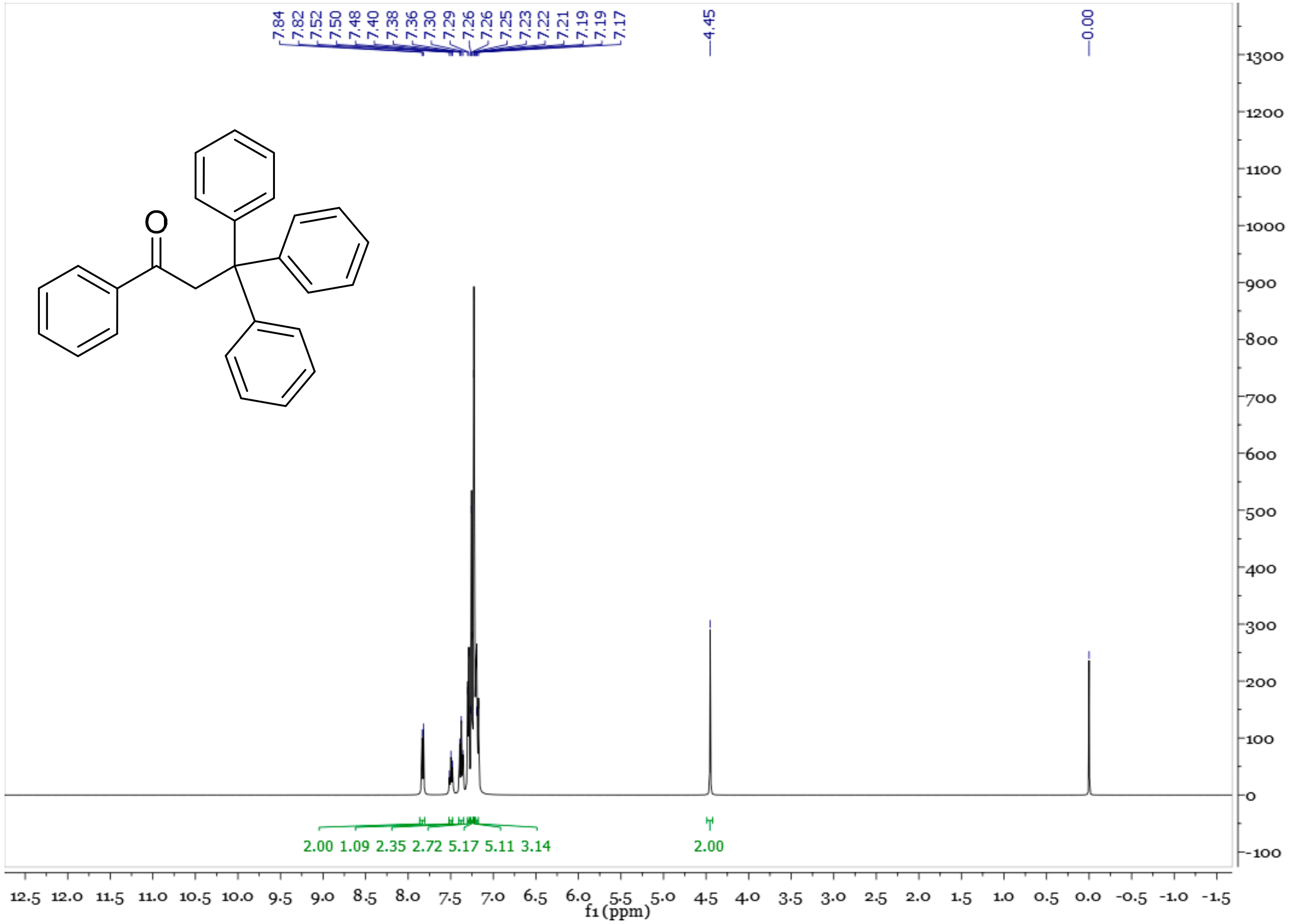

^{13}**C NMR** (101 MHz, $CDCl_3$) δ 197.24, 147.33, 147.07, 132.87, 131.31, 130.16, 129.58, 129.41, 128.55, 128.15, 128.01, 127.94, 127.53, 126.21, 49.53, 31.10
Same as reported in Koppolu, S. R.; Naveen, N.; Balamurugan, R., Triflic Acid Promoted Direct α-Alkylation of Unactivated Ketones Using Benzylic Alcohols via in Situ Formed Acetals. *J. Org. Chem.* **2014,** *79* (13), 6069-6078.

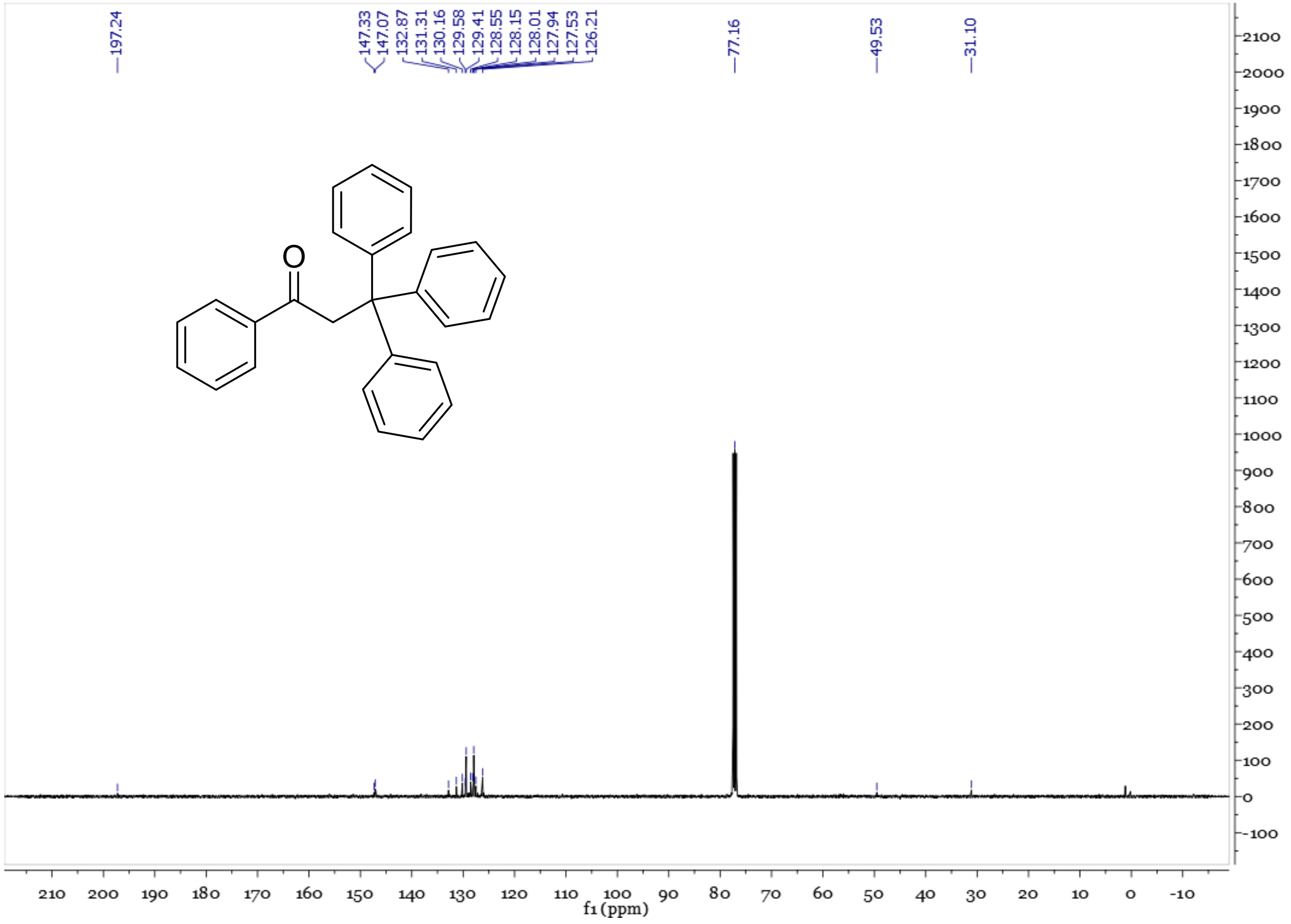

¹H-NMR (400 MHz, Chloroform-*d*) δ 7.81 (d, *J* = 7.3 Hz, 2H), 7.36 (t, *J* = 7.3 Hz, 1H), 7.30-7.10 (m, 8H), 7.06 (t, *J* = 7.2 Hz, 1H), 6.98 (t, *J* = 7.6 Hz, 2H), 6.87 (t, *J* = 7.3 Hz, 1H), 4.38 – 4.25 (m, 2H), 1.01 (d, *J* = 4 Hz, 3H). Same as reported in Jayamani, M.; Pant, N.; Ananthan, S.; Narayanan, K.; Pillai, C. N., Synthesis of indenes from phenylpropanones using alumina catalyst. *Tetrahedron* **1986,** *42* (15), 4325-4332.

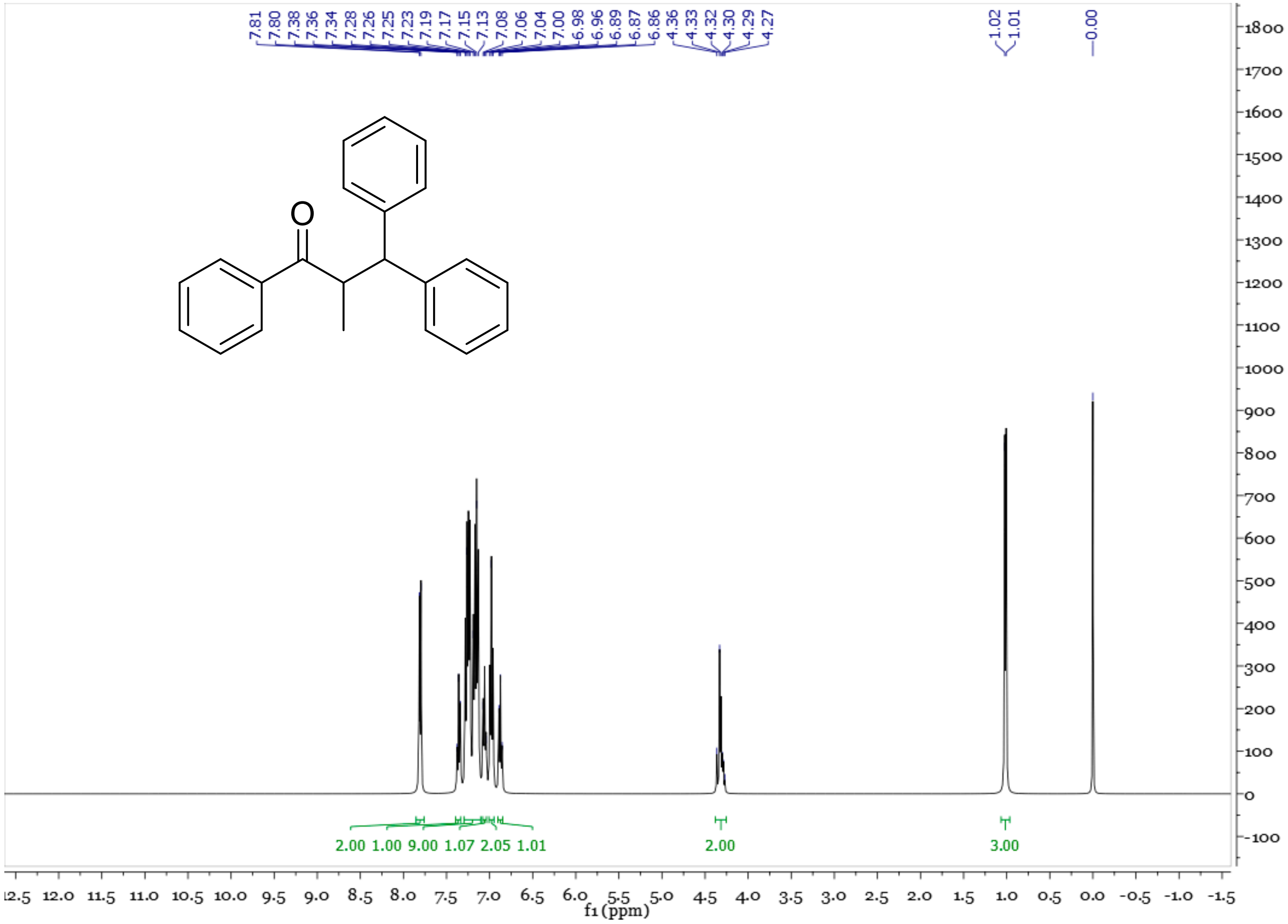

13C NMR (101 MHz, Chloroform-*d*) δ 203.37, 143.60, 143.08, 136.75, 133.00, 128.68, 128.66, 128.52, 128.45, 128.13, 127.68, 126.54, 126.18, 54.32, 44.79, 18.07. Same as reported in Jayamani, M.; Pant, N.; Ananthan, S.; Narayanan, K.; Pillai, C. N., Synthesis of indenes from phenylpropanones using alumina catalyst. *Tetrahedron* **1986,** *42* (15), 4325-4332.

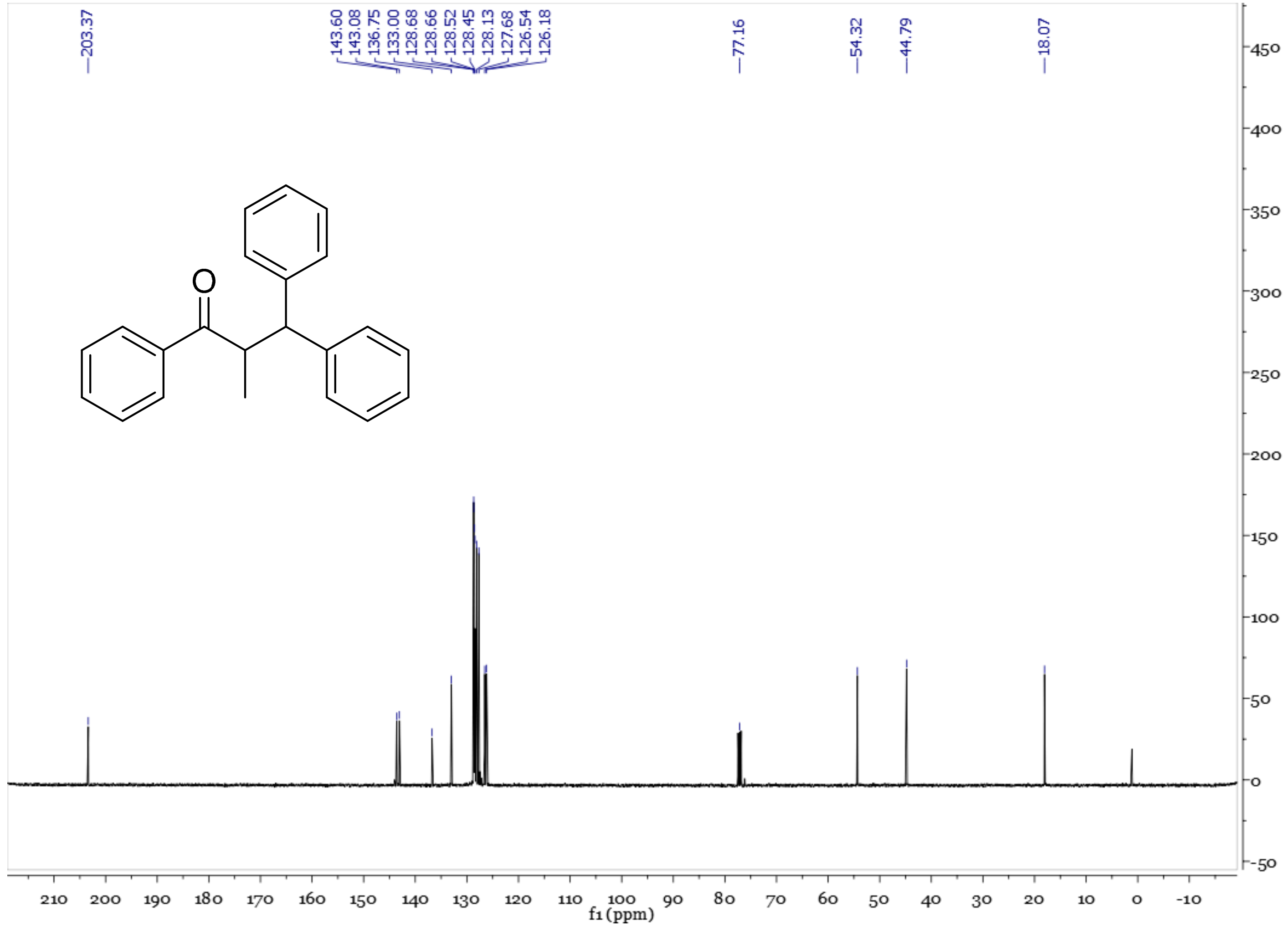

^{1}H-NMR (400 MHz, Chloroform-*d*) δ 7.95 (d, *J* = 7.2 Hz, 2H), 7.49 (t, *J* = 7.4 Hz, 1H), 7.39 (t, *J* = 7.6 Hz, 2H), 7.29 – 7.23 (m, 5H), 7.20 (m, 4H), 7.05 (m, 6H), 4.67 (q, *J* = 6.8 Hz, 1H), 1.52 (d, *J* = 6.9 Hz, 3H). IR (ATR): 3083.9, 3058.3, 3024.9, 2972.9, 2927.9, 2870.2, 2853.2, 1681.3, 1220.25, 698.9, 607.6 cm^{-1} .HRMS (ESI-MS) m/z: [M +H]+ Calcd $(C_{28}H_{24}O)H^+$ 377.1900; Found 377.1905. colorless viscous oil

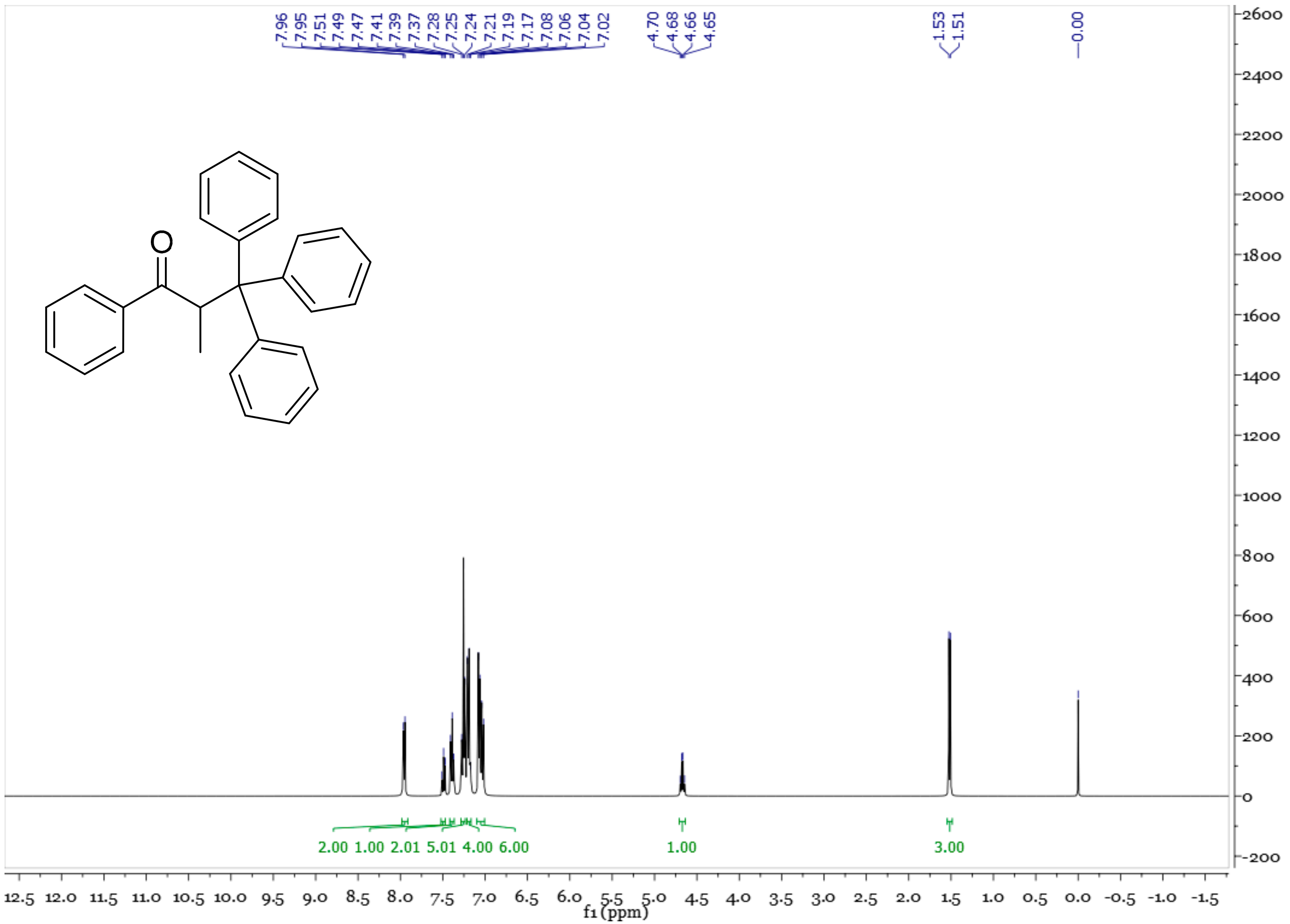

^{13}C NMR (101 MHz, Chloroform-*d*) δ 200.55,143.89, 142.56, 139.41, 136.60, 132.93, 130.06, 129.54, 128.94, 128.61, 128.42, 127.83, 126.44, 77.16, 56.57, 47.43, 19.64.

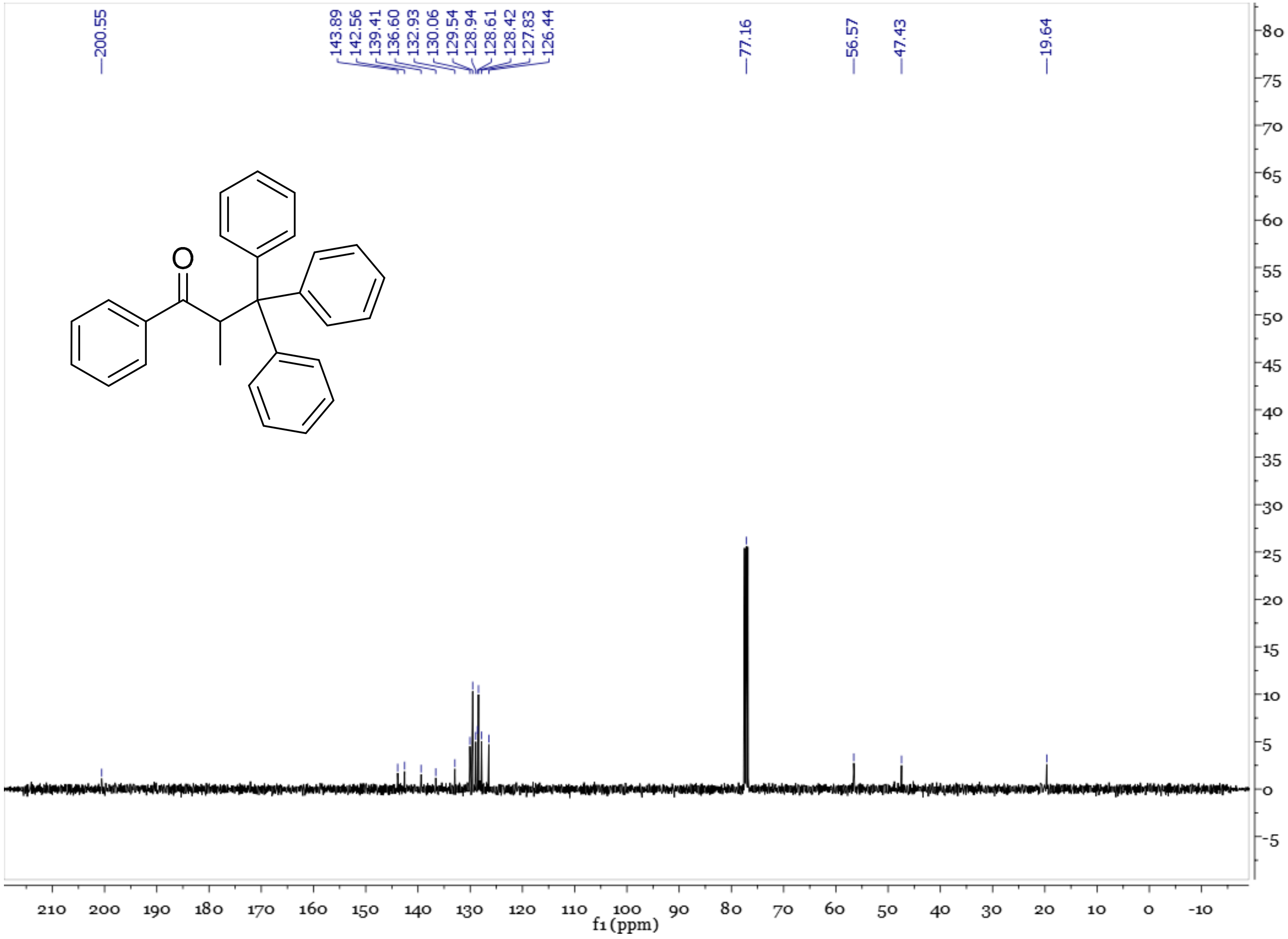

Mass Spectrum

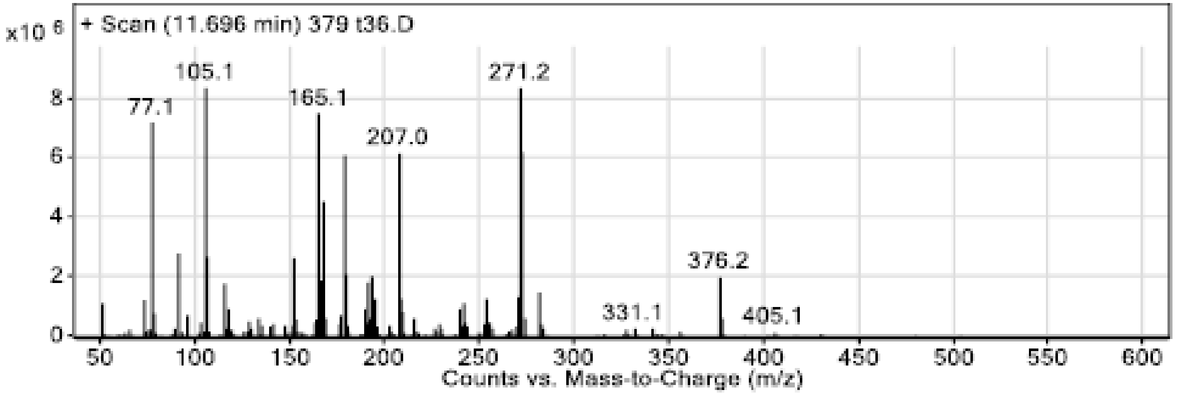

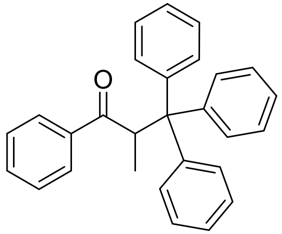

MW= 376g/mol

¹H-NMR(400 MHz, Chloroform-*d*) δ 7.41 (d, *J* = 8.6 Hz, 2H), 7.35 – 7.19 (m, 3H), 7.12 (m, 3H), 7.00 (d, *J* = 6.4 Hz, 2H), 2.97 (s, 2H), 1.19 (s, 6H). same as reported in Barluenga, J.; Aguilar, E.; Olano, B.; Fustero, S., Mild and regiospecific reduction of masked 1,3-dicarbonyl derivatives to monocarbonyl compounds and primary and secondary amines. *J. Org. Chem.* **1988,** *53* (8), 1741-4.

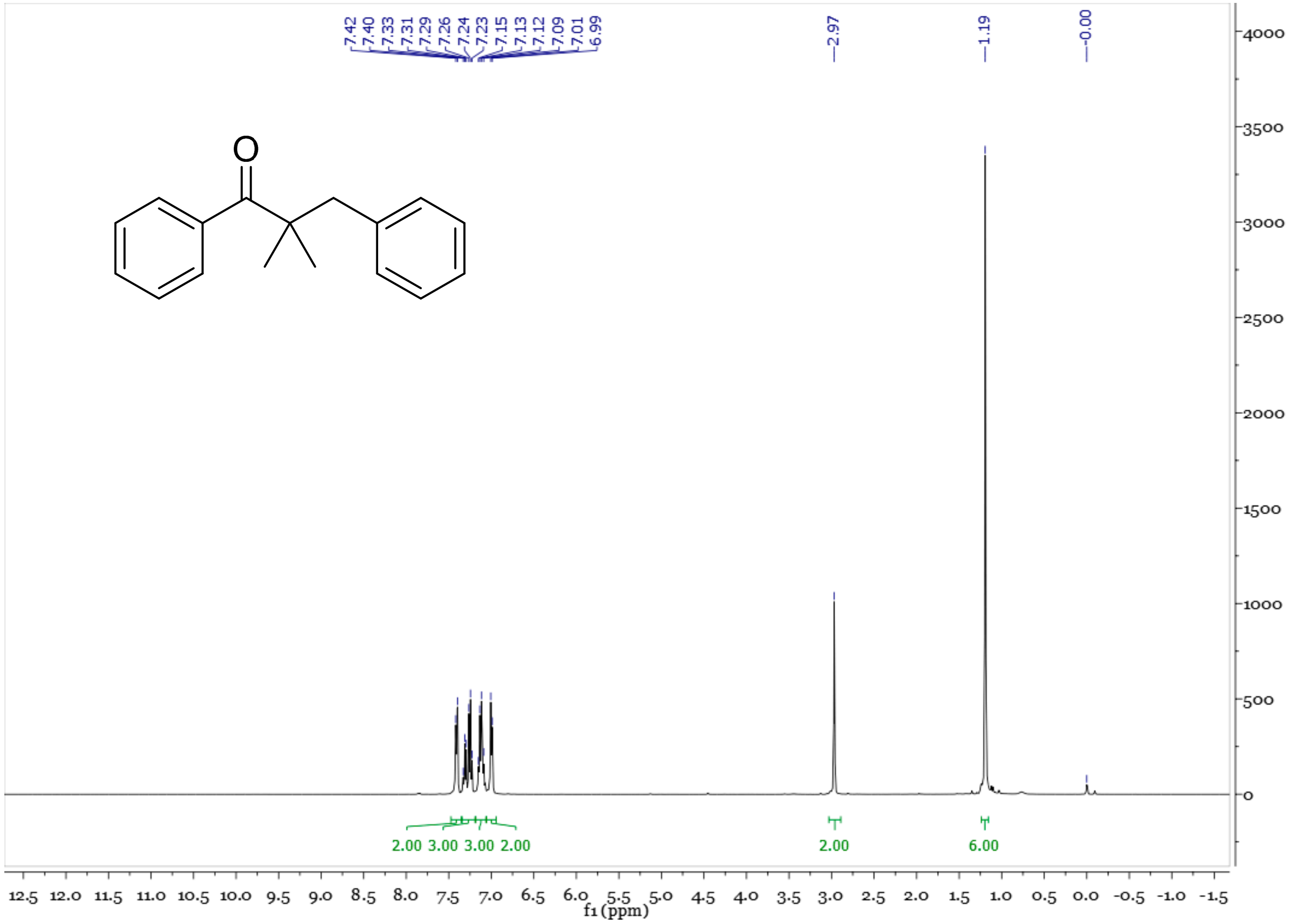

^{13}C NMR (101 MHz, Chloroform-*d*) δ 209.44, 139.44, 137.89, 130.66, 130.52, 128.06, 127.49, 126.44, 48.78, 46.25, 26.11. Same as reported in Barluenga, J.; Aguilar, E.; Olano, B.; Fustero, S., Mild and regiospecific reduction of masked 1,3-dicarbonyl derivatives to monocarbonyl compounds and primary and secondary amines. *J. Org. Chem.* **1988,** *53* (8), 1741-4.

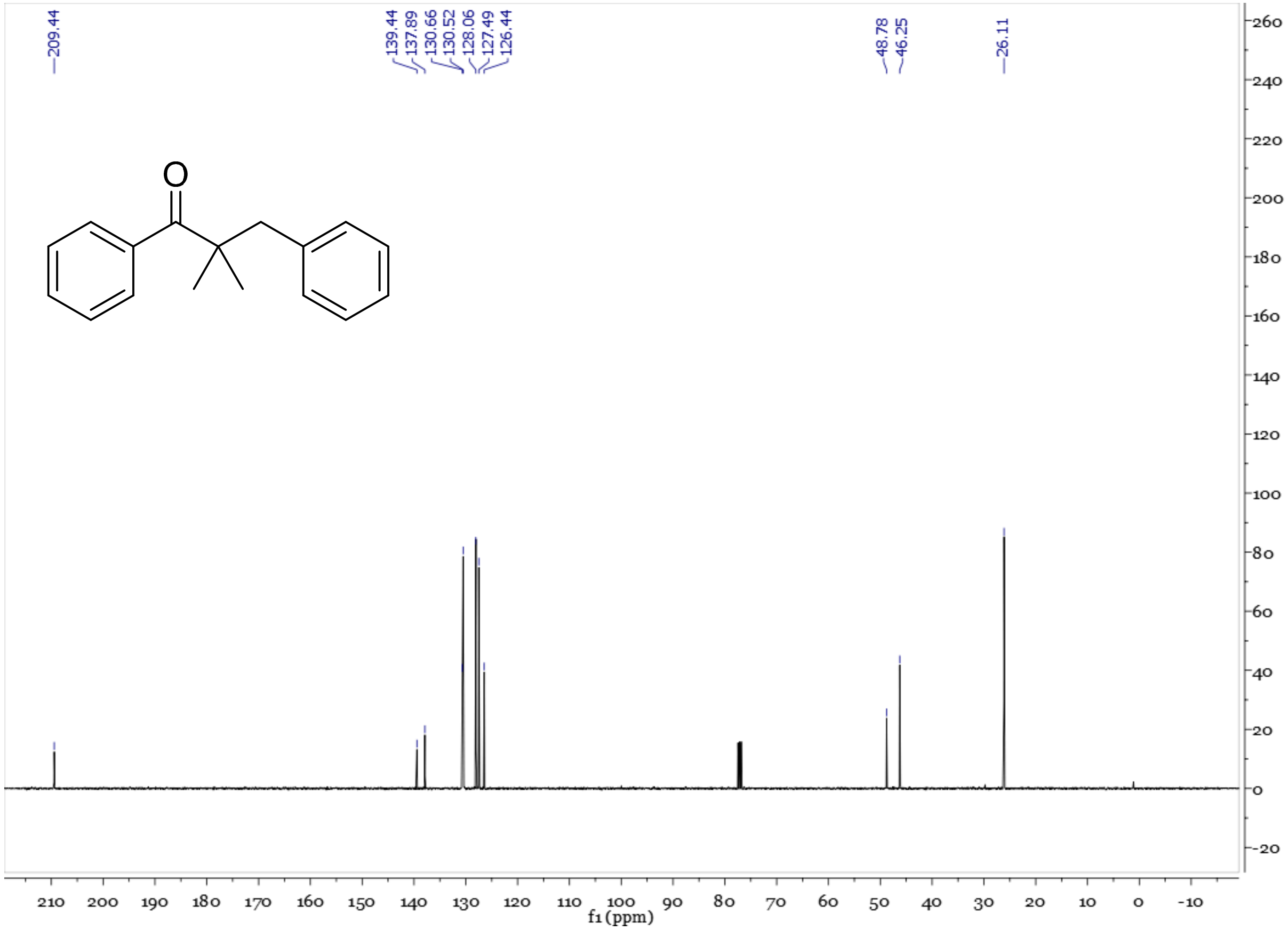

^{1}H-NMR ^{1}H NMR (400 MHz, Chloroform-*d*) δ 7.28 (t, *J* = 7.0 Hz, 1H), 7.22 – 7.14 (m, 12H), 7.10 (t, *J* = 7.0 Hz, 2H), 4.66 (s, 1H), 1.34 (s, 6H). Same as reported in Chen, W.; Liu, Z.; Tian, J.; Li, J.; Ma, J.; Cheng, X.; Li, G., Building Congested Ketone: Substituted Hantzsch Ester and Nitrile as Alkylation Reagents in Photoredox Catalysis. *J. Am. Chem. Soc.* **2016,** *138* (38), 12312-12315.

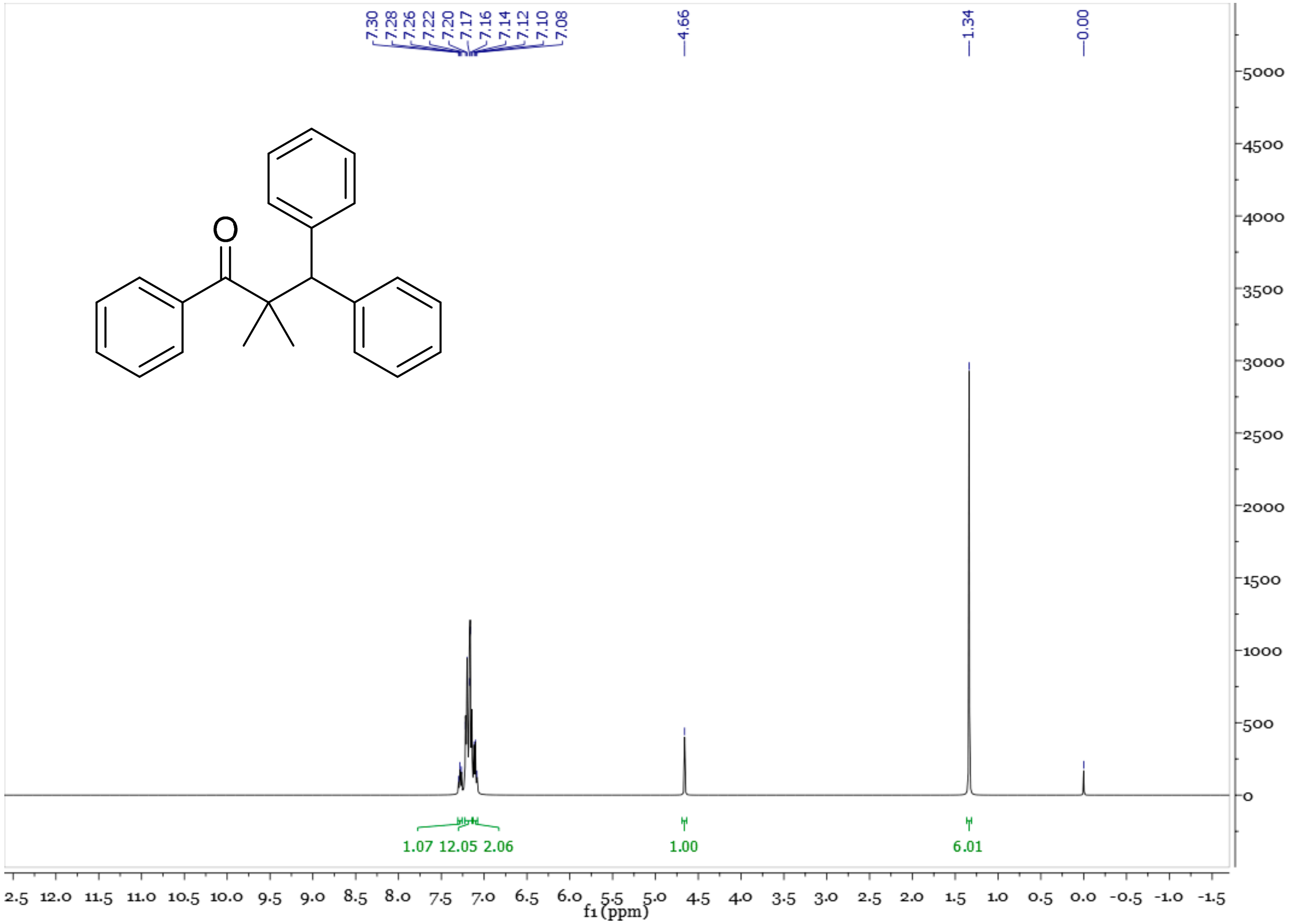

^{13}C NMR (101 MHz, Chloroform-*d*) δ 210.65, 141.68, 140.21, 130.33, 128.22, 127.97, 127.23, 126.56, 58.77, 52.19, 25.71. Same as reported in Chen, W.; Liu, Z.; Tian, J.; Li, J.; Ma, J.; Cheng, X.; Li, G., Building Congested Ketone: Substituted Hantzsch Ester and Nitrile as Alkylation Reagents in Photoredox Catalysis. *J. Am. Chem. Soc.* **2016,** *138* (38), 12312-12315.

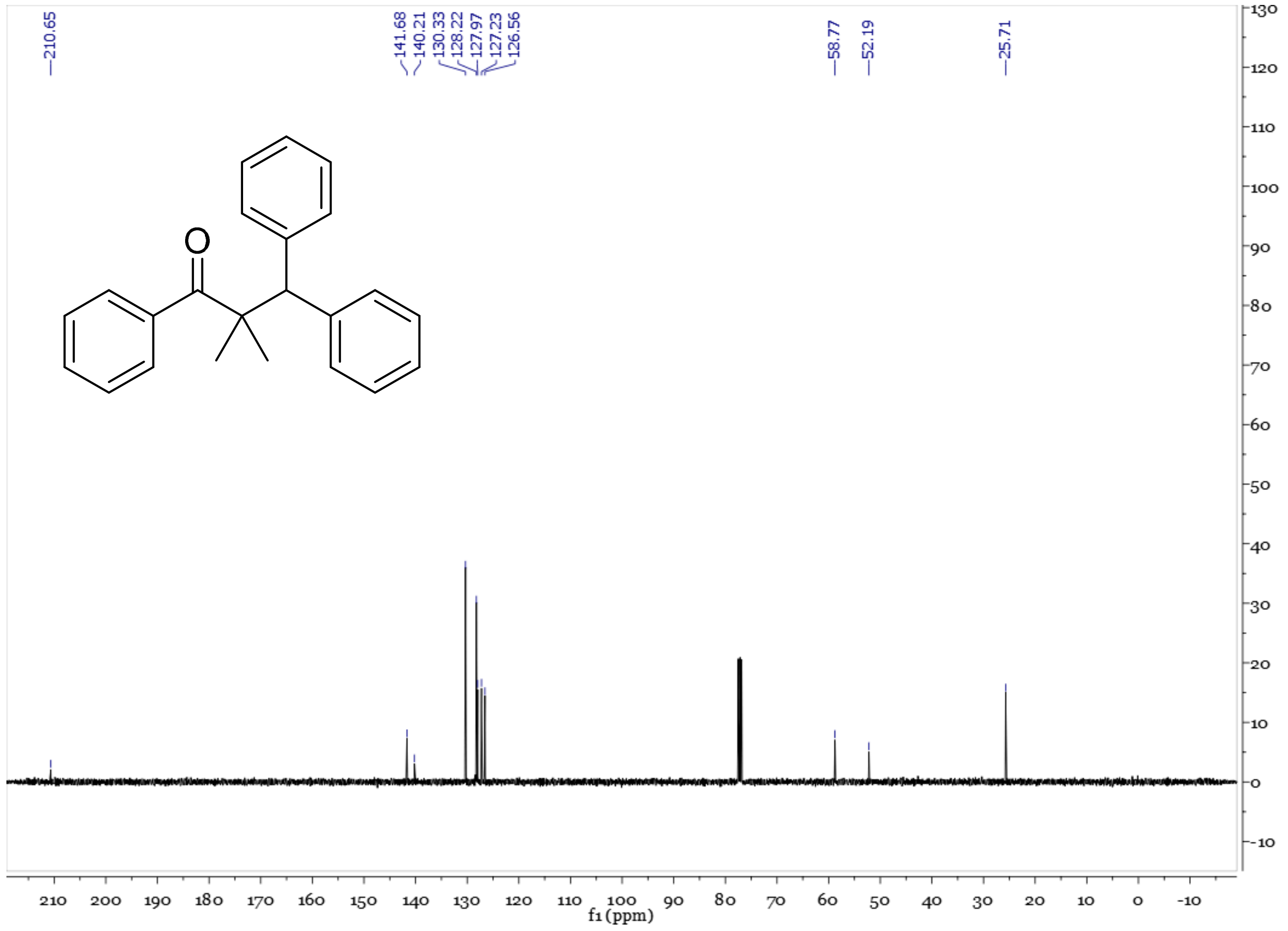

Mass Spectrum

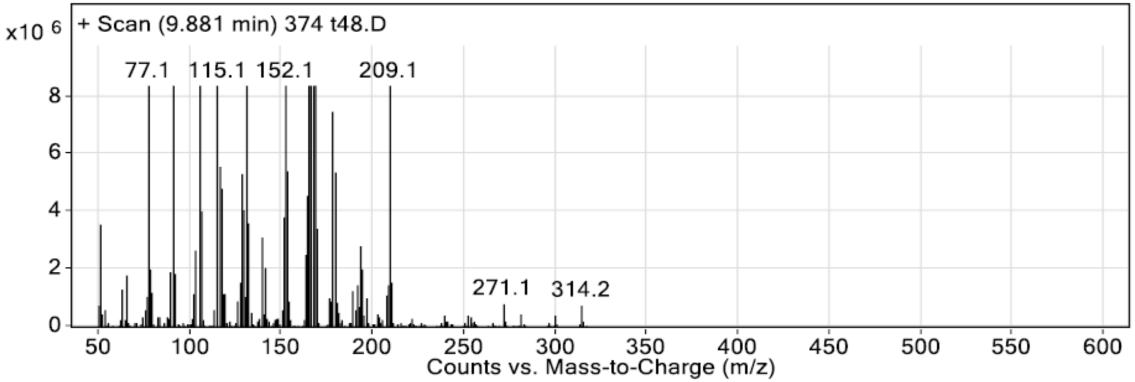

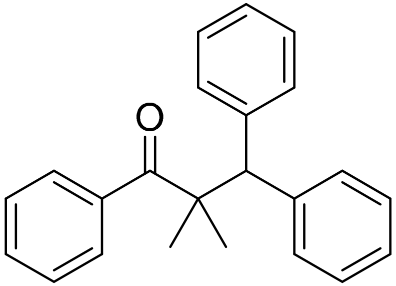

MW= 314g/mol

^{1}H-NMR (400 MHz, Chloroform-*d*) δ 7.49 (d, *J* = 7.2 Hz, 2H), 7.37 (t, *J* = 7.4 Hz, 1H), 7.25 (m, 11H), 7.09 (d, *J* = 6.9 Hz, 6H), 1.58 (s, 6H). IR (ATR): 3083.9, 3058.3, 3054.9, 2961.7, 2927.4, 2854.3, 1674.7, 1264.2, 734.6, 704.1 cm^{-1} HRMS (ESI-MS) m/z: [M + Na]+ Calcd for $(C_{29}H_{26}O)H^{+}$ 391.2056; Found 391.2062. colorless viscous oil

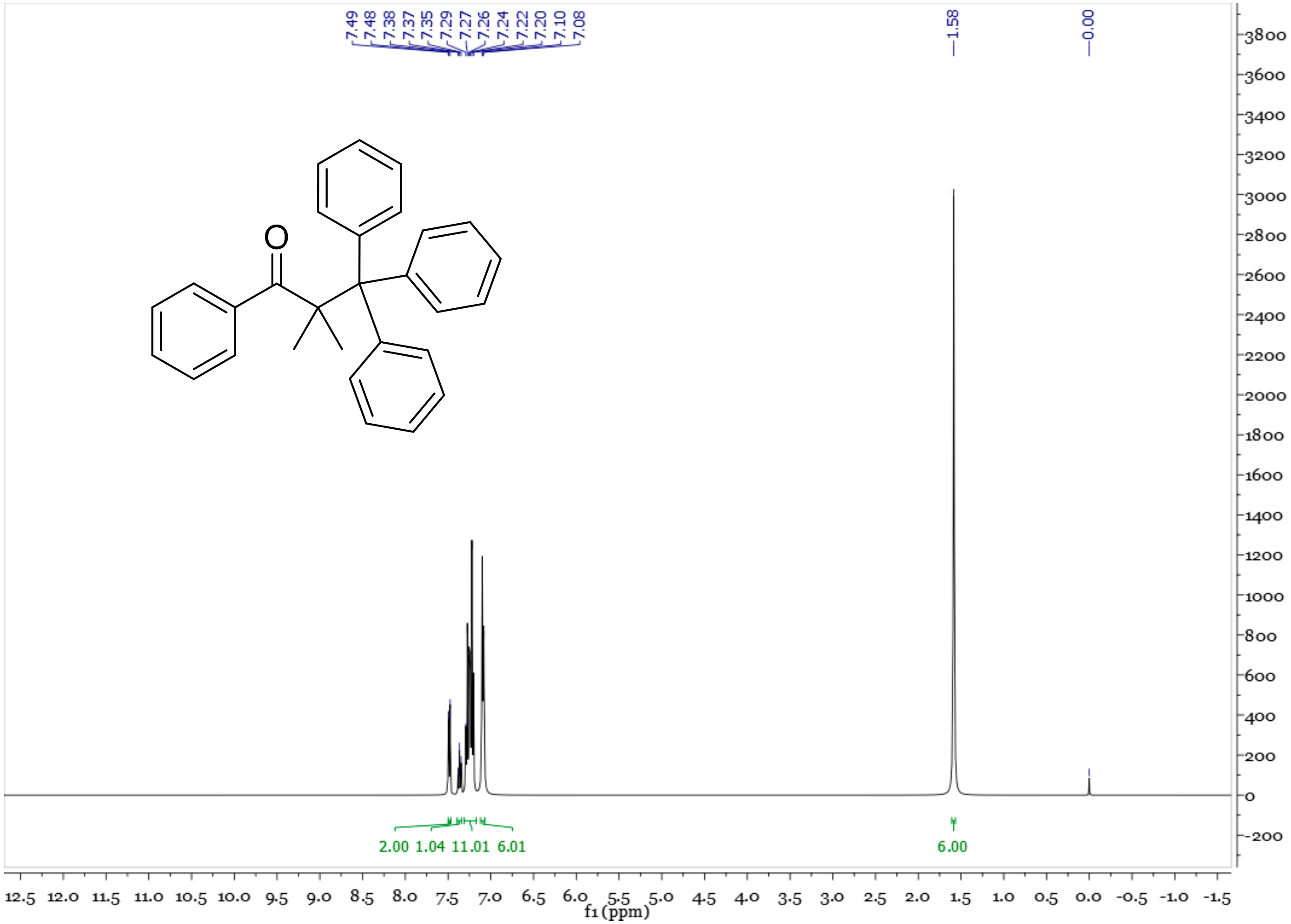

(59) ^{13}C NMR ^{13}C NMR (101 MHz, Chloroform-*d*) δ 203.80, 143.88, 143.35, 142.45, 136.35, 131.76, 130.10, 129.88, 129.52, 128.44, 127.99, 126.48, 125.87, 56.47, 51.23, 27.87.

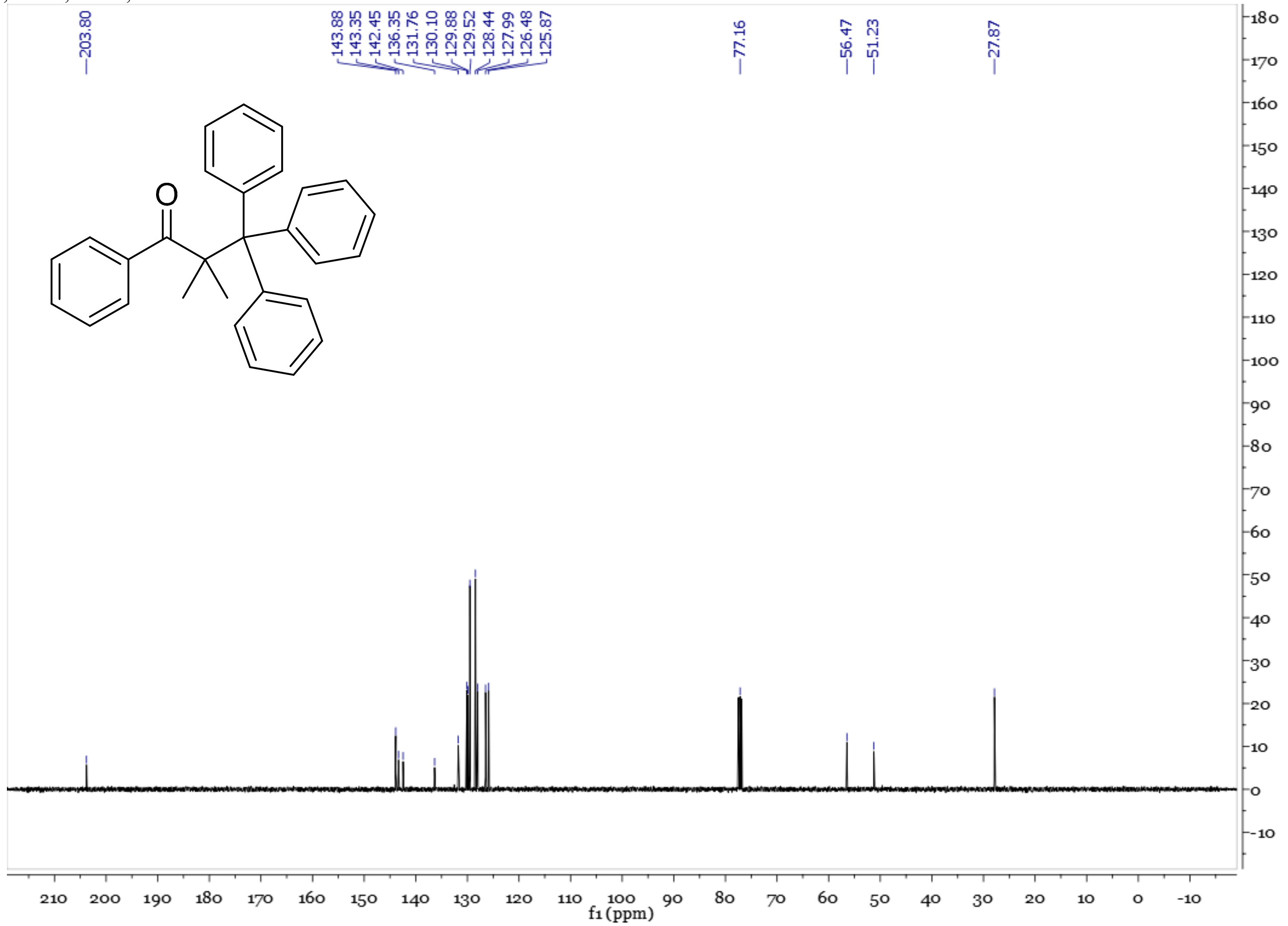

Mass Spectrum

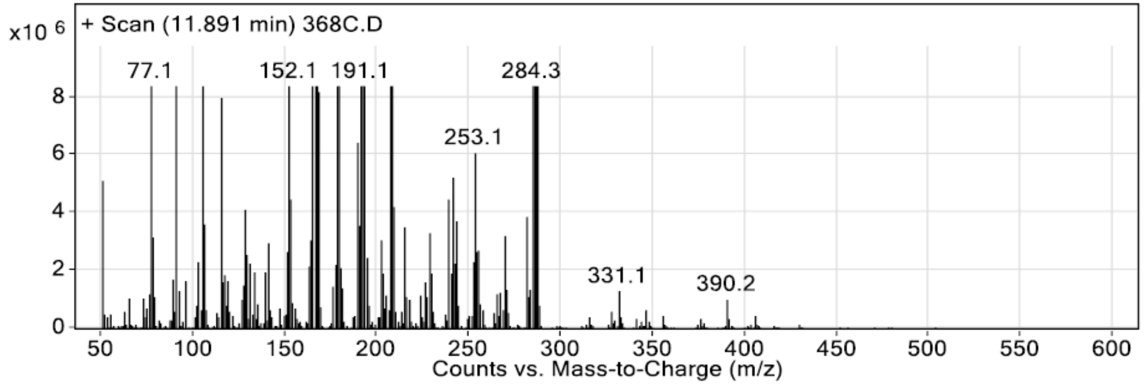

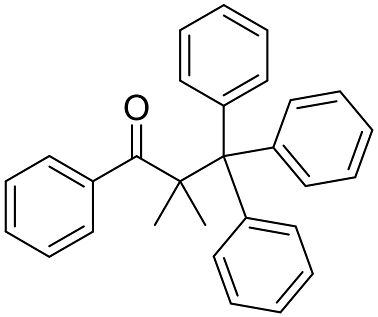

MW= 390g/mol
